# Establishment of emerging practices and research priorities for telerehabilitation in solid organ transplantation: meeting report and narrative literature review

**DOI:** 10.3389/fresc.2025.1535138

**Published:** 2025-03-28

**Authors:** Dmitry Rozenberg, Sherrie Logan, Sahar Sohrabipour, Nicholas Bourgeois, Anita Cote, Robin Deliva, Astrid De Souza, Rienk de Vries, Maoliosa Donald, Manoela Ferreira, Donna Hart, Megha Ibrahim Masthan, Tania Jaundis-Ferreira, Sandrine Juillard, Michael Khoury, Afsana Lallani, Diana Mager, Istvan Mucsi, Ani Orchanian-Cheff, Jennifer L. Reed, Puneeta Tandon, Karthik Tennankore, Elaine Yong, Lisa Wickerson, Sunita Mathur

**Affiliations:** ^1^ Toronto Lung Transplant Program, Ajmera Transplant Centre, University Health Network, Toronto, ON, Canada; ^2^ Division of Respirology, University of Toronto, Toronto, ON, Canada; ^3^ Canadian Donation and Transplantation Research Program (CDTRP), Edmonton, AB, Canada; ^4^ Temerty Faculty of Medicine, University of Toronto, Toronto, ON, Canada; ^5^ Lung Transplant Program, Centre Hospitalier de L’Université de Montréal, Montreal, QC, Canada; ^6^ School of Human Kinetics, Trinity Western University, Langley, BC, Canada; ^7^ Department of Pediatrics, British Columbia Children’s Hospital Research Institute, Vancouver, BC, Canada; ^8^ Department of Rehabilitation Services, Hospital for Sick Children, Toronto, ON, Canada; ^9^ Cumming School of Medicine, University of Calgary, Calgary, AB, Canada; ^10^ Division of Respirology, University Health Network, Toronto, ON, Canada; ^11^ School of Physical & Occupational Therapy, McGill University, Montreal, QC, Canada; ^12^ Department of Microbiology, Infectiology and Immunology, Faculty of Medicine, Université de Montréal, Montreal, QC, Canada; ^13^ Department of Microbiology, CHUM Research Center (CRCHUM), Montreal, QC, Canada; ^14^ Department of Pediatrics, University of Alberta, Edmonton, AB, Canada; ^15^ Department of Agricultural, Food and Nutritional Sciences, Dept of Pediatrics, University of Alberta, Edmonton, AB, Canada; ^16^ Ajmera Transplant Centre, and Division of Nephrology, University Health Network, Toronto, ON, Canada; ^17^ Division of Nephrology, University of Toronto, Toronto, ON, Canada; ^18^ Library and Information Services, University Health Network, Toronto, ON, Canada; ^19^ University of Ottawa Heart Institute, Ottawa, ON, Canada; ^20^ School of Epidemiology and Public Health, Faculty of Medicine, University of Ottawa, Ottawa, ON, Canada; ^21^ School of Human Kinetics, Faculty of Health Sciences, University of Ottawa, Ottawa, ON, Canada; ^22^ Department of Medicine, Division of Gastroenterology (Liver Unit), University of Alberta, Edmonton, AB, Canada; ^23^ Department of Medicine, Division of Nephrology, Dalhousie University, Halifax, NS, Canada; ^24^ Department of Physical Therapy, University of Toronto and Ajmera Transplant Centre, University Health Network, Toronto, ON, Canada; ^25^ School of Rehabilitation Therapy, Queen’s University, Kingston, ON, Canada; ^26^ Rehabilitation Sciences Institute, University of Toronto, Toronto, ON, Canada

**Keywords:** transplantation, rehabilitation, exercise, physical activity, telerehabilitation

## Abstract

Solid organ transplantation (SOT) is a life-saving procedure for those with end-stage organ dysfunction. The main goals of SOT are to improve quality of life and daily function, which are supported by pre- and post-transplant rehabilitation. In-person rehabilitation programs have traditionally been the standard-of-care for delivering rehabilitation for SOT patients. Many programs have adopted a virtual delivery model [telerehabilitation (TR)], an approach that has become increasingly used given restrictions to in-person delivery during the COVID-19 pandemic. Presently, TR programs are being used both clinically and in research with variable practices. A 2-day virtual meeting held in February 2023 brought together over 30 Canadian adult and pediatric researchers, clinicians, and patient and family partners across SOT. The meeting objectives were: (1) To facilitate knowledge exchange and dialogue in TR between patient partners, healthcare professionals, researchers, and key stakeholders, and (2) Identify gaps in clinical practice and research in TR. The discussion focused on delivery methods of TR, digital tools, facilitators and barriers of TR, and the effects of TR on physical and mental health in both adult and pediatric populations. This meeting report incorporates a narrative literature review of SOT and rehabilitation articles in the last 20 years. Future directions in TR are highlighted leading to the development of key research priorities targeted towards improved delivery of TR in SOT patients.

## 1 Introduction

Transplantation is a well-established procedure for end-stage organ disease known to improve health-related quality of life (HRQL) and survival (1, 2). In 2022, a total of 2,936 solid organ transplants (SOT), including adult and pediatric kidney, liver, heart, lung, and pancreas, were performed in Canada (3) and a total of 42,887 SOT in the United States (4). However, SOT candidates have impairments in functional capacity that does not return to predicted levels post-transplantation resulting in reduced physical activity levels and impairments in HRQL (5–7).

Traditional facility-based rehabilitation programs not only help with recovery in exercise capacity, muscle strength, and HRQL post-transplant, but also mitigate physical deconditioning pre-transplant (8–10). The level of physical function pre- and post-transplant is associated with physiological benefits (improved skeletal muscle function, bone density, and metabolic factors) (11–13), shorter hospitalizations (14), lower surgical complications (15) and hospital readmissions (16), and improved post-transplant survival (16, 17). However, facility-based programs have faced challenges with respect to accessibility and uptake as we emerge from the COVID-19 pandemic with increased in-person activities, which have accelerated telerehabilitation (TR) initiatives (18–20).

Telerehabilitation programs have emerged as a promising alternative to in-person rehabilitation programs in SOT populations (21–25), which is the delivery of rehabilitation through various telecommunication strategies, such as videoconferencing, phone calls, or internet applications (26). TR can enhance access to medically underserviced populations (27–29), reduce financial and time constraints for patients geographically distant from a transplant centre (30, 31) and may help improve accessibility (28, 32).

There is emerging evidence on the feasibility and effectiveness of TR programs on adult and pediatric patient outcomes with several ongoing trials (33–36). Despite limited evidence, TR was rapidly adopted during the COVID-19 pandemic with a strong positive response from the patient partner community. The optimal structure of TR programs such as individual versus group training, synchronous versus asynchronous, and the balance of virtual and in-person assessments remains unclear. Further, practical aspects of exercise progression, technological support and equipment needed for safe physiological monitoring and effective delivery of TR pre- and post-transplant in adult and pediatric populations requires additional investigation. Other challenges in the field of TR include patient and provider digital literacy, digital access in rural areas, data privacy and governance (37, 38). Thus, there are several questions related to training, technological support and implementation that require ongoing study in SOT (30).

To more thoroughly understand the delivery of TR in SOT, a virtual meeting was held to identify important facilitators, barriers, and care priorities for rehabilitation for adult and pediatric transplant candidates, recipients, caregivers, and healthcare providers. The objectives of the meeting were: (1) To facilitate knowledge exchange and dialogue between patient and family partners, clinicians, researchers, and other key partners as it relates to TR, and (2) Identify gaps in clinical practice and research in TR to improve delivery of TR in SOT patients.

## 2 Methods

A two-day virtual meeting titled “Telerehabilitation in Solid Organ Transplantation in Canada: Celebrating Achievements and Designing the Future” was held through Zoom in February 2023. The meeting was held in English for 5-hours each day with over 30 attendees, including researchers, healthcare providers, adult and pediatric patient partners, and caregivers. A pre-meeting with the principal applicants and co-applicants of the Canadian Institute of Health Research funded grant was held in October 2022 to finalize the list of participants, speakers, and the meeting agenda (Supplementary Appendix A1). Details on invitation of meeting participants can be found in Supplementary Appendix A2.

The meeting included 3 presentations by patient partners regarding their transplant and rehabilitation experiences, and 18 presentations by researchers, clinical investigators, and patient partners on key adult (*n* = 13) and pediatric (*n* = 5) topics in TR, including the evolving landscape of TR, delivery methods, user experiences with TR, technological considerations, and clinical applications of TR tools, Supplementary Appendix A1. Facilitated, small group discussions were held in breakout rooms, then larger group discussions with all attendees, and interaction among attendees using Google Jamboard (a tool that allowed attendees to write their reflections in a shared document). The meeting was recorded for reference and a summary of the discussion is outlined in Supplementary Appendix A2.

A literature search was conducted by a librarian to identify key papers in the field using Ovid Medline on June 24, 2024. A combination of key words and subject headings for SOT and rehabilitation, including various study designs such as review articles, cross-sectional or cohort studies were searched to ensure key papers were included. To maximize the literature included in our narrative review, studies had to report on at least one aspect of TR or some consideration of rehabilitation delivery in SOT or chronic disease populations. Only studies with full-text available in English and published in the last 20-years were included, Supplementary Appendix A3. Case reports were excluded.

## 3 Results

**Figure 1:**
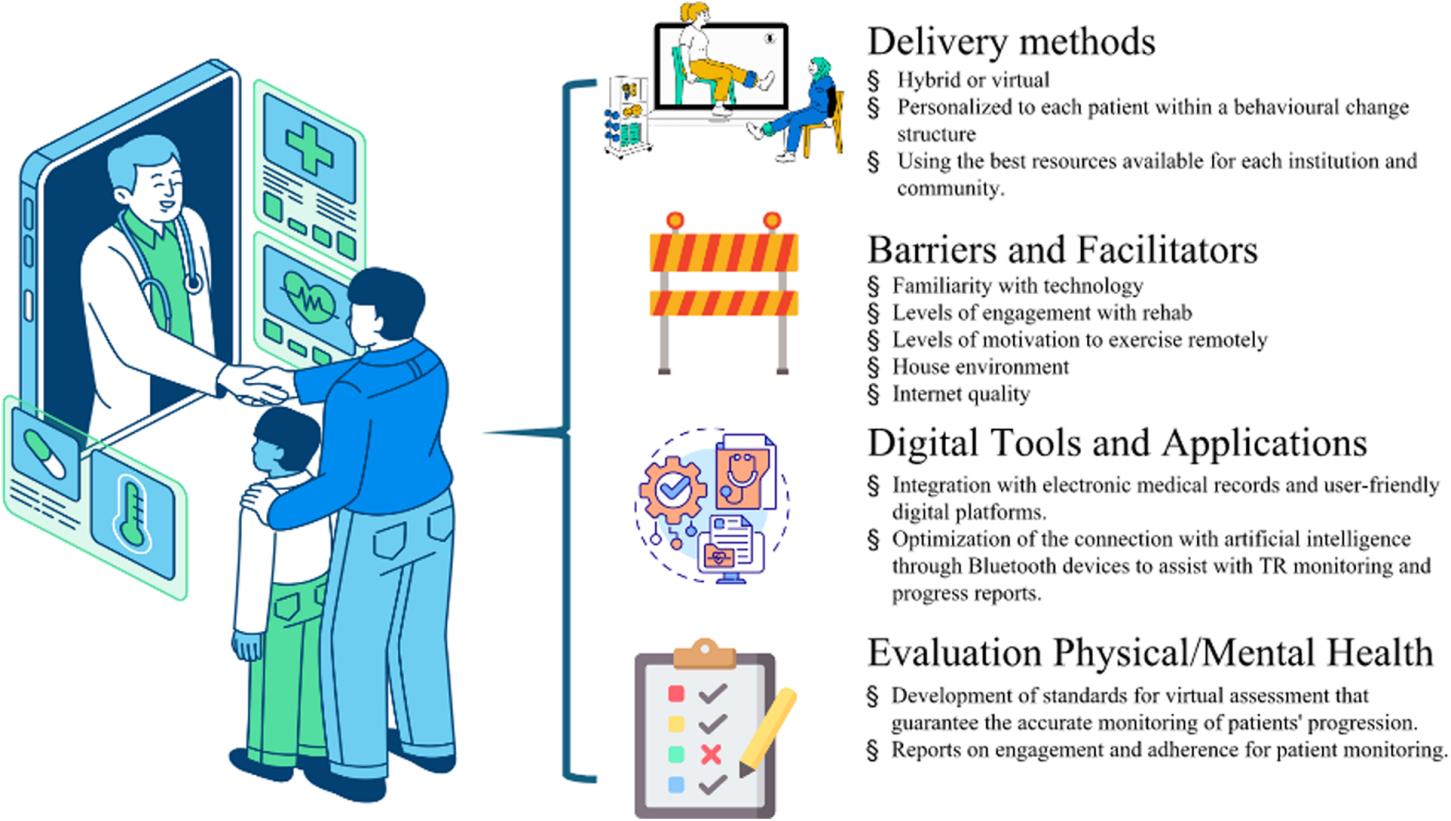
Aspects that should be considered for successful telerehabilitation in solid organ transplantation. Credits from the left and top to bottom: Reproduced with permission from “Lined Isometric Online Pediatric Consultation” by M. Wallflower; “Expressive Lined Virtual Healthcare” by Dianne Rosario; “Isolated Construction Barrier Flat Design” by Iconsy, licensed under Free Content License. Reproduced with permission from “2D Customizable Thin Line Icon Health Records Access Concept” by bsd studio; “Evaluation” by Uniconlabs, licensed under Pro Content License. Figure was generated using stock images from Canva.

The results from the meeting are synthesized and grouped into four main topics: (1) Delivery methods and safety of TR; (2) Digital tools and available applications; (3) Barriers and facilitators to TR delivery; and (4) Evaluation of physical and mental health with exercise, as in Figure 1. The topics pertained to both adult and pediatric SOT candidates and recipients with a dedicated pediatric section are summarized below.

### 3.1 Delivery methods and safety of telerehabilitation

Several models of TR delivery were discussed at the meeting, specifically synchronous (i.e., direct observation) and asynchronous programs, optimal timing of TR implementation and duration, balance between in-person and virtual visits, equipment, and digital requirements. A variety of videoconference platforms (i.e., MS Teams, Zoom, Vivify Health) (21, 35, 39) have been utilized across TR with good uptake. Furthermore, technological considerations of TR include access to a computer or portable electronic device with potential video capability, access to the internet and wearable or ancillary devices.

Safety considerations were discussed at the meeting. Specifically, several programs undertook a hybrid approach where the initial assessment was conducted on site followed by training using TR. The ability to perform synchronous monitoring during exercise and having an emergency action plan (e.g., phone nearby, caregiver present at home, doors unlocked in case emergency personnel need entry) were highlighted as important safety considerations, especially with higher risk transplant populations such as those with cardiovascular disease (40), or those with higher falls risk (41). The participant's ability to access technical support or an alternative contact number for the healthcare provider was highlighted as a key safety aspect. An environmental safety survey of the home environment, either done virtually or as a home visit, to evaluate falls risk (e.g., loose rugs, appropriate chair height/stability) was suggested (42). It was also suggested that patients who experience hospitalizations or significant changes in health status be re-evaluated by a healthcare professional prior to re-starting their program.

### 3.2 Digital tools and applications

Digital applications can be utilized to facilitate TR. These applications can capture changes in exercise routines and adherence given possible setbacks such as infections, hospital admissions and musculoskeletal injuries (43). Some adult SOT recipients expressed a desire to be able to share their exercise data with healthcare providers, their peers or family members.

Healthcare providers expressed that they wanted tools that were evidence-based, had the availability of an exercise library, and the ability to integrate these exercises with digital applications. Furthermore, proficiency in using digital tools, adequate training, and ability to use the tools at multiple transplant sites were highlighted as important priorities by healthcare providers (44). Healthcare providers and researchers expressed the importance of integrating the patient perspective in designing, testing and implementing these digital applications.

A web-based platform (Heal-Me) was presented as an example of a multidisciplinary program developed with input from patients to support nutrition and exercise programming through videoconferencing for individuals with chronic conditions, including SOT patients (45, 46). An updated version has also been adapted to provide mental health support, including mind-body movement and chronic disease skills management (47, 48). Another program developed for chronic kidney disease patients (Kidney BEAM) (49, 50) provides an opportunity for participants to join live classes or virtual groups to chat with patients and providers, and more importantly, create a sense of community with benefits in both physical and mental health (51).

Generally, most patient partners reported a positive experience after participating in TR. Specifically, they enjoyed using physical activity trackers, such as Fitbits, which increased their motivation to exercise and served as a potential incentive to participate in TR programs offering Fitbits. Further, patients stated that they enjoyed being able to complete exercises at home instead of on-site, as it allowed them to save time and money on travel.

### 3.3 Barriers and facilitators to telerehabilitation delivery

A number of perspectives on TR were discussed during the meeting. The emphasis on TR throughout the transplant journey was an important consideration in the SOT population. It was highlighted that TR should be personalized, flexible in terms of delivery options, and be able to combine both physical and mental health support. Several facilitators and barriers are described below.

#### 3.3.1 Facilitators

Facilitators to TR were highlighted during the meeting, Table 1. Specifically, the ability to tailor the program to the unique needs and desires of patients, accounting for the variability that may exist in preferences for individual versus group exercises. Secondly, involving patients when developing digital applications with consideration of visual, hearing and language challenges was discussed. The ability to provide mental health support through TR was echoed as an important strategy for overall well-being. Furthermore, having a “transplant mentor,” described as an SOT recipient who has gone through the process, may prove to be beneficial in providing motivational support through their lived experiences (52). Other facilitators to TR included having strong multidisciplinary support, well developed educational programs with effective and safe exercises, involvement of caregivers with TR support, and providing incentives (i.e., the ability keep a physical activity tracker after use). Also, focusing on the transition period from hospital to home allows in-person rehab to be performed initially followed by TR at home.

**Table 1:** Facilitators and barriers of telerehabilitation (TR) discussed at the meeting.

Theme	Facilitators	Barriers
Accessibility	• Can reach more patients, especially those who live far from rehabilitation centres • Reduces commuting/parking costs • Provide adequate training and education on using various tools	• Not all patients have access to the technology/devices required to participate in TR, and/or have a high-speed, reliable internet connection • Some platforms may be challenging to learn for patients and providers • Hearing and visual impairments • Lack of staff and funding, especially to provide long-term TR
Safety	• Home-visit can improve safety by ensuring safe space to exercise • Safety protocols, guidelines, and education session • Can prevent spread of infectious diseases especially in immunocompromised patients	• No safety protocols exist on how to best monitor patients during TR, or what to do in the event of an emergency • Lack of in-person assessments could hinder care • Might be difficult to learn initial exercises virtually for the first time • Patients may be too sick to participate
Support and mental health	• Providing mental health support during TR • Having a “transplant mentor” or peer-to-peer support • Caregivers can help overcome barriers and increase motivation • Improves continuity of care and builds stronger relationships with healthcare team • Develop a transplant community among patients and providers • Organizations in community could also be trained to provide support (e.g., fitness professionals at gyms)	• Some support and mental health counselling may be more conducive to in person assessments
Communication	• Method for patients and providers to be able to communicate based on patient preference (e.g., secure messaging service, phone calls, live options during TR)	• Lack of online communication skills may limit the patient's ability to use all of the possible options available with TR
Transition period	• In-person demonstration of exercises prior to patient's discharge from hospital could help with learning and safety prior to initiating TR • Baseline assessment can help track efficacy of programs	• Some digital and exercise equipment may need to be obtained/purchased by participant before starting TR • Consideration of TR utilization during transitions from pediatrics to adult care
Patient preferences/individualization	• Asking patients about their preferences (e.g., group vs. alone, hybrid vs. virtual) can improve retention and satisfaction • Incorporate fun exercises and activities, especially for pediatric patients, as well as incentives for participating	• TR programs that are not individualized may not be appropriate for all patients’ unique needs and goals, which could reduce engagement • Individualized programs would be more costly and require more staff
Language	• Patients who have language barriers can still participate in virtual sessions with the help of a caregiver	• Fewer language options for providing TR
Motivation and engagement	• Patients who prefer virtual sessions due to the comfort of training from their home might find the sessions motivating and engage better	• Patients might not want to engage or continue with TR • Some patients might prefer group sessions while others prefer individual training • Some patients may not be familiar with available TR programs
Caregivers	• Caregiver presence can increase safety during TR • Caregiver engagement in TR programs can enhance SOT patient motivation	• Parents may prevent their children from participating in TR due to fear of injury • Some patients require a caregiver/second person present during TR, which creates additional barriers with availability

#### 3.3.2 Barriers

Some drawbacks of TR included challenges in learning the physical exercises virtually, the possibility that some programs may not be able to provide a personalized approach, and decreased motivation with perceived loss of community with an asynchronous program, and potential restrictions with internet accessibility in some rural communities (53). The safety, validity and adaptation of several in-person assessments to the remote environment (i.e., six-minute walk test) should be an area for further investigation (54). Additional barriers to TR include lack of technological access, potential language barriers, and program level factors, as shown in Table 1.

Some research programs provided participants with tablets if needed to facilitate access to TR (55). Potential risk factors that may warrant closer supervision, including the potential need for facility-based programs, include the presence of unstable heart disease, fluctuations in anticoagulation levels, limited digital literacy, and presence of frailty (56, 57). Motivation was also cited as an important consideration with some recipients keen to minimize healthcare visits post-transplant. Furthermore, it remains unclear whether certain physiological outcomes can be achieved in a similar manner (i.e., target heart rate zones) with TR as with in-person exercises. Table 2 provides several research gaps identified with TR.

**Table 2:** Future research priorities and questions identified to address gaps in telerehabilitation (TR).

Considerations in telerehabilitation	Research questions
Safety and monitoring	• What information should be included in safety guidelines for patients and providers engaging in TR (e.g., live monitoring to call 911 in case of an emergency, educating patients on concerning symptoms)? • How can patients be best assessed and monitored remotely? • What are the best approaches for studies using TR to systematically report any adverse events?
Mental health	• How can mental health support be incorporated for patients during the pre- and post-transplant period (e.g., counselling, peer-to-peer support with others experiencing a similar journey)?
Technology	• What platform(s) should TR be delivered to patients that is accessible, user-friendly, and secure? • What wearable devices can patients use (e.g., smart watches, pulse oximeters) to accurately and equitably track and record biometric data (e.g., heart rate, oxygen saturation)? • How can patient information collected remotely be incorporated into the clinical workflow in an efficient and secure manner?
Delivery	• What factors are important to consider when deciding how to deliver rehabilitation (e.g., virtual, in-person, or hybrid)? • Should initial assessments be done in-person or virtually? • How can individualized programs (instead of a one-size-fits-all approach) be developed to meet patients’ unique goals and needs?
Timing	• When should TR be initiated during patient's transplant journey? • How often should TR be delivered pre- and post-transplant? • How long should TR continue post-transplant?
Collaboration and knowledge translation	• What is the best approach for centres and programs to share resources and technological experiences with TR to improve patient outcomes?
Motivation and engagement	• How to make TR engaging and motivational for patients?

### 3.4 Evaluation of physical and mental health

The evaluation of physical and mental well-being in the virtual environment were discussed. The need for an in-person assessment was addressed as several physical assessments (i.e., six-minute walk test) and clinical impressions (i.e., frailty) have more in person standardized procedures. However, frailty assessments have been carried out in transplant candidates virtually and may be an important baseline assessment for assessing reversibility (58). Patient reported outcome measures can be completed on various electronic platforms and may provide useful information related to physical functioning, psychological symptoms, and overall physical and mental health (59, 60). In fact, TR creates an opportunity to provide rehabilitation to vulnerable or frail transplant patients who may not have been able to participate in a center-based program due to travel or physical limitations. Furthermore, wearable devices (i.e., physical activity trackers, oximeters, daily activity logs) may provide an opportunity to guide exercise training and monitor progress throughout TR, but need to consider that some of these devices are not medical grade and may lack accuracy (61). The integration of mental health and nutritional supports in TR may have a synergistic benefit with exercise training. The group felt that evaluating the contribution of TR on physiological benefits (i.e., exercise capacity, strength, balance) and clinical outcomes (i.e., hospitalization data, readmissions, infections, graft function) may be helpful in understanding its effects on health-care utilization, as there is a paucity of literature in this area (Table 2).

### 3.5 Telerehabilitation in the pediatric SOT population

The specific benefits, challenges and research questions for the pediatric SOT population were also discussed. Telerehabilitation programs for pediatric transplants have demonstrated improvements in strength and self-confidence at the end of a 12–16 week resistance program (24). However, researchers identified recruitment and program enjoyment as challenges among youth (25). Breakout session discussions and presentations by youth and clinicians reinforced the importance of individualizing the program based on the developmental age of the child to promote enjoyment, improve adherence and enhance motivation. Depending on the age of the individual, other discussions to increase enjoyment included family involvement, peer support, games or activities with rewards and virtual reality. For example, an ongoing randomized crossover feasibility trial of a video-game linked TR exercise platform (known as MedBIKE), which is currently underway for 10–18 year old heart transplant recipients was discussed (36). Adolescence was highlighted as a period of rapid change in personal growth that may hinder motivation to participate (62, 63).

Physical literacy was highlighted by researchers and clinicians during the workshop as an important component to develop positive physical activity habits, exercise self-efficacy and promote pediatric neurodevelopment (64). However, workshop participants also cautioned that too much focus on the educational benefits of physical activity within a TR program could reduce the pleasure for youth. Thus, a greater emphasis on physical literacy may help promote enjoyment in physical activity (65). One clinician indicated that the pre-transplant phase is ideal for TR recruitment, as families are eager to minimize frailty and health consequences before surgery (8, 66). Furthermore, pre-transplant TR can help improve confidence with physical activity and prepare patients and families for the post-transplant period.

Parental concerns regarding safety of exercise and physical activity post-transplant are potential barriers that may require mitigation strategies for TR programs (67, 68). However, caregivers participating in the workshop mentioned that their confidence in safety improved as they observed their children participating in sporting activities as previously described in liver transplant recipients (67). Parental and sibling participation in TR was observed to be important in improving adherence in liver recipients (69). However, caregiver mental health barriers, such as post-traumatic stress disorder, anxiety or depression related to their child's transplant journey, may require additional preventative support for families pre-transplant in integrating physical activity (67, 68). Moreover, pediatric SOT recipients typically have a much more heterogeneous array of pre-transplant diagnoses. Thus, each child must be considered individually with respect to safety and ability to participate in various TR programs. Age- and ability-specific individualizations are essential to ensure the appropriate delivery of TR for pediatric SOT recipients.

Assessment tools, including wearables (oximeters, fitness activity trackers) (70), quality of life and fatigue scales (PedsQL 4.0, PedsQL Multidimensional Fatigue Scale) (71, 72), physical activity questionnaire (PAQ-C or A) (73), musculoskeletal strength [Bruininks-Oseretsky Test of Motor Proficiency (BOT-2) (74), functional testing, FitnessGram] and communication platforms (WelTel, Zoom) (75, 76) were identified by researchers, clinicians and patients/caregivers as useful to monitor health, performance, changes in fitness and activity levels and engagement in TR programs. However, if patient families are required to absorb costs associated with assessment tools (wearables, online programs), they may be a barrier for lower-income families and equitable access is an important consideration (43).

A number of important knowledge gaps remain regarding TR in pediatric SOT recipients. First, there is a paucity of randomized control trials evaluating the safety and effectiveness of exercise delivery. In addition, various modalities of exercise programs have not been adequately compared in pediatric participants such as high-intensity interval training which has been shown to be superior to moderate intensity continuous exercise in adult heart transplant recipients (77). While pediatric transplant recipients are known to be quite sedentary with suboptimal physical activity levels (78), barriers to increasing activity and the potential for TR programs to have sustained improvements in activity and self-efficacy require further study.

## 4 Discussion

By creating an environment for communication and knowledge exchange during our two-day virtual meeting with SOT patient partners, caregivers, clinicians, and SOT rehabilitation researchers, we were able to identify key research questions and priorities in the field of TR with the goals of improving transplant outcomes and patient-centered research within a public healthcare system. The meeting discussion focused on delivery methods of TR, digital tools, facilitators and barriers of TR, and effects of TR on physical and mental health in both adult and pediatric populations.

Several models of TR were discussed at the meeting that have been adopted in the last several years. Most clinical and research TR programs combine a hybrid approach with in person and virtual assessments and training (79, 80). The optimal balance between in-person and virtual assessment and training remains unclear, but also depends on the needs of patients and transplant center resources. Furthermore, the timing of TR in the post-transplant period and how to utilize the transition period in hospital to engage patients on some of the technological and equipment requirements for TR remain to be determined. In addition, it is important to account for the non-linear trajectories in transplant populations, given frequent infections, hospital admissions, and management of underlying comorbidities (81–83). TR holds a great deal of promise as telehealth has been shown in many chronic disease populations to improve treatment adherence, increase the ability to capture measurements, and promote self-management (57, 84). TR also offers an opportunity for greater accessibility to exercise professionals who are familiar with SOT patients and their needs, which was a common barrier expressed by SOT recipients related to physical activity resources (43).

There are a number of digital health tools that are publically available and those that have been developed by individual clinical and research programs. These technological advancements hold promise with integration into TR programs as they can provide a broad range of physiological monitoring (i.e., heart rate, oxygen), nutritional support, information on organ-related function, and optimal strategies for informing the healthcare team (43). Furthermore, digital resources should be complementary and provide flexibility in selecting required features for TR accounting for the variable health changes in SOT recipients. Digital health applications can help promote greater self-monitoring and independence among patients pre and post-SOT, which can aid TR programs in monitoring their patients virtually (44).

The clinical sustainability of TR programs is an important consideration. Specifically, consideration for TR utilization beyond the immediate training period or the optimal integration of digital applications and tools still needs to be defined. The two-day meeting highlighted variability in both clinical and research practices in the delivery of TR (virtual vs. in-person training), degree of supervision (synchronous vs. asynchronous), and number of participants (individual vs. group classes). Given the evolving field of TR in the last few years, there are no existing guidelines that provide guidance on the integration of TR or telehealth for SOT recipients (85). Furthermore, there remain questions regarding start-up costs for programs and patients as it relates to exercise equipment, digital resources, and health-care personnel availability. The funding availability for TR programs remains to be defined and is certainly variable across SOT programs and geographic locations.

The need for developing standardized safety protocols for TR was highlighted to ensure that appropriate guidelines are in place for both patients and providers. The common elements among ongoing studies evaluating the feasibility and safety of TR in SOT are as follows: (1) having an emergency action plan in place (2) education on technical support as needed (3) environmental safety survey, and (4) support of caregivers (34, 35, 79). Reassuringly, no serious adverse events have been reported with TR in several SOT populations (21, 24, 33, 86). Future work in the area of TR is needed to evaluate and create safety guidelines in the delivery and evaluation of TR outcomes in SOT.

Another important priority that was identified throughout the two-day meeting was the need for mental health support for SOT candidates and recipients and for these resources to be integrated into TR during both the pre- and post-transplant period to help with adherence and adaptation of new health conditions. Previous research found that structured exercise itself had positive effects on mental health for patients both pre- and post-transplant (80, 87); however, the need for formal mental health support beyond physical exercise was emphasized, such as through counselling and peer support. In addition, the timing and progression of TR in the post-transplant period needs to be considered in the context of the transplant experience given the variability in both physical and emotional experiences leading up to the transplant, peri-operative period, and functional recovery for patients, which will influence readiness to participate in physical activity and exercise training (43). In addition, the availability of some of these supports for caregivers of transplant recipients was articulated by several stakeholders during the meeting, given some of the emotional challenges that caregivers may experience. Furthermore, peer support for caregivers and transplant patients was expressed as one potential strategy to assist with mental health challenges.

The association between participation in TR with pre- and post-transplant clinical outcomes such as hospitalizations and mortality have not been evaluated. However, there is evidence in transplant recipients that low physical HRQL in kidney recipients is associated with reduced survival (88, 89). In addition to physical health, mental and general well-being have been shown to be associated with graft and overall survival in renal transplant recipients (13, 88, 90). In liver transplant recipients, exercise is associated with improved exercise capacity, physical function, and HRQL, but long-term sustainability and association with clinical outcomes remain unclear (20). Thus, TR may help identify patients at higher risk of functional decline and potentially provide earlier opportunities for intervention through rehabilitation. However, the optimal timing and duration of TR pre and post-transplantation remains to be defined.

There are a number of research questions in TR that were identified at the meeting (Table 2). There is an increased need to evaluate the optimal delivery strategy for TR. Specifically, a focus is needed on the actual delivery of TR, including optimal monitoring strategies, integration of TR into electronic medical records, and development of flexible digital platforms (e.g., applications or web-based tools) that are sustainable and up-to-date with the ability to evaluate the quality of TR delivery. Potential areas for future research include creating flexible TR programs that can be personalized, performing economic evaluation of preventative lifestyle factors that could be facilitated with TR, or using virtual reality (91) and/or artificial intelligence (AI) to enhance TR, and assist with TR recommendations. To date, there have been no studies on the use of virtual reality or AI in TR for SOT patients, therefore highlighting an exciting area of future research in this patient population, especially among adolescent and young adult SOT recipients. Digital health applications that incorporate multifaceted health domains such as exercise guidance, mental health supports, nutrition, organ specific features, and the capability of sharing information with family, peers and healthcare providers require further evaluation. Importantly, collaboration among SOT TR programs, researchers, clinicians, and patient partners was identified as a future priority to enhance knowledge mobilization and engagement in collaborative research opportunities. These collaborations would allow sharing of resources and experiences to improve TR and patient outcomes.

Even though not discussed at the 2-day meeting, it is important to highlight several future considerations in TR related to cost-effectiveness, healthcare provider training and policy considerations. TR has been shown to be feasible and helpful in providing increased access to rehabilitation across a number of chronic conditions in adult and pediatric populations relative to traditional in-person rehabilitation practices (92). There have been only several studies that have evaluated cost of TR in cardiac, respiratory and musculoskeletal settings, all of which have favoured TR over standard rehabilitation (93–97). However, to our knowledge the cost-effectiveness or long-term sustainability of TR in SOT candidates or recipients has not been evaluated to-date despite the sudden implementation during the COVID-19 pandemic (21, 98). Further, the training of healthcare providers, as it relates to online communication and technological skills, has been shown to be an important factor in healthcare provider uptake, satisfaction, and implementation of TR (92, 99). It is important that organizations utilizing TR have the appropriate technological infrastructure (i.e., equipment, standardized platforms) and informatics support for both providers and patients. This is a key consideration from a health policy sustainability standpoint that funding is available for organizations to provide appropriate technological training and support for effective TR delivery for providers, patients and caregivers (100), highlighted in Supplementary Table S2. Based on the literature (92, 100), many of the facilitators and barriers are common across chronic conditions including the SOT population. However, the optimal delivery structure of TR in SOT populations (balance of in-person vs. virtual program), funding for infrastructure support, and cost-effectiveness requires further evaluation. There are several considerations in the SOT population such as greater travel distance from healthcare center, increased infectious concerns with immunosuppression, and frequent clinical appointments that may favor TR compared to other chronic or surgical conditions.

There are similar limitations worth highlighting. Firstly, the views in this report were from participants affiliated with the CDTRP with diverse representation across SOT centers, organ types, adult and pediatric populations, and varied experience with TR; however, the views expressed may differ across jurisdictions and healthcare settings. Second, we utilized a narrative literature review without a critical analysis of the included articles; thus, limiting our ability to comment on study validity. Lastly, the 2-day virtual meeting did not discuss cost-effectiveness or implementation in low or middle income countries, which are important considerations in future studies.

## 5 Conclusion

Telerehabilitation has emerged as an important intervention post COVID-19 pandemic in the SOT population. A few TR models of care have been applied both in the clinical and research settings across SOT recipients, but further research with regards to their cost, effectiveness, optimal delivery and association with clinical outcomes is needed. Several important considerations for TR in transplantation were identified in the meeting. The use of digital applications, wearables, and health tools is promising in facilitating TR, but further refinement on organ specific and lifestyle needs will need to be considered. The sustainability of TR beyond the immediate training period, along with multidisciplinary resources such as mental health and nutritional support, were highlighted as key resources for TR effectiveness. Future work will need to explore opportunities for increased clinical and research collaboration across centers in TR delivery, digital applications, and collaborative funding resources to develop the required evidence for TR sustainability.

## Data Availability

The original contributions presented in the study are included in the article/Supplementary Material, further inquiries can be directed to the corresponding author.
