# Supplementary material for: Establishment of emerging practices and research priorities for telerehabilitation in solid organ transplantation: meeting report and narrative literature review
Source: Front Rehabil Sci. 2025 Mar 28;6:1535138. doi: 10.3389/fresc.2025.1535138 (PMC11985446; doi:10.3389/fresc.2025.1535138)
Supplement: Supplementary file 1 [file Datasheet1.pdf]

Supplementary Material

Establishment of Emerging Practices and Research Priorities for Telerehabilitation in Solid Organ Transplantation: Meeting Report and Literature Review

Dmitry Rozenberg[1,2], *Sherrie Logan[3], Sahar Sohrabipour[4], **Nicholas Bourgeois[5], Anita Cote[6,7], Robin Deliva[8], Astrid De Souza[7], Rienk de Vries[3], Maoliosa Donald[9], Manoela Ferreira[3], Donna Hart[3], Megha Ibrahim Masthan[10], Tania Jaundis-Ferreira[11], Sandrine Juillard[12,13], Michael Khoury[14], Afsana Lallani[3], Diana Mager[15], Istvan Mucsi[16,17], Ani Orchanian-Cheff,[18] Jennifer L. Reed[19,20,21], Puneeta Tandon[22], Karthik Tennankore[23], Elaine Yong[3], Lisa Wickerson[1,24], *Sunita Mathur[25]

1. Toronto Lung Transplant Program Ajmera Transplant Center, University Health Network, Toronto, Ontario
2. Division of Respirology, University of Toronto, Toronto, Ontario
3. Canadian Donation and Transplantation Research Program (CDTRP), Edmonton, Alberta
4. Temerty Faculty of Medicine, University of Toronto, Toronto, Ontario
5. Lung Transplant Program, Centre Hospitalier de l'Université de Montréal, Montreal, Quebec
6. School of Human Kinetics, Trinity Western University, Langley, British Columbia
7. British Columbia Children's Hospital Research Institute, British Columbia
8. Department of Rehabilitation Services, Hospital for Sick Children, Toronto, Ontario
9. Cumming School of Medicine, University of Calgary, Calgary, Alberta
10. Division of Respirology, University Health Network, Toronto, Ontario
11. School of Physical & Occupational Therapy, McGill University, Montreal, Quebec
12. Department of Microbiology, Infectiology and Immunology, Faculty of Medicine, Université de Montréal, Montreal, Quebec
13. CHUM Research Center (CRCHUM), Montreal, Quebec
14. Department of Pediatrics, University of Alberta, Edmonton
15. Department of Agricultural, Food and Nutritional Sciences, Dept of Pediatrics, University of Alberta, Edmonton
16. Ajmera Transplant Centre, and Division of Nephrology, University Health Network, Toronto, Ontario
17. Division of Nephrology, University of Toronto, Toronto, Ontario
18. Library and Information Services, University Health Network, Toronto, ON, Canada
19. University of Ottawa Heart Institute, Ottawa, Ontario
20. School of Epidemiology and Public Health, Faculty of Medicine, University of Ottawa, Ottawa, Ontario
21. School of Human Kinetics, Faculty of Health Sciences, University of Ottawa, Ottawa, Ontario
22. Department of Medicine, Division of Gastroenterology (Liver Unit), University of Alberta, Edmonton, Alberta
23. Department of Medicine, Division of Nephrology, Dalhousie University, Halifax, Nova Scotia
24. Department of Physical Therapy, University of Toronto and Ajmera Transplant Centre, University Health Network, Toronto, Ontario
25. School of Rehabilitation Therapy, Queen's University, Kingston, Ontario and Rehabilitation Sciences Institute, University of Toronto, Toronto, Ontario

Supplemental Material

	Pages
**Supplementary Appendix 1: Meeting Agenda**	**3-4**
**Supplementary Appendix 2: Description of Facilitated Discussions**	**5 -6**
**Supplementary Appendix 3: Literature Search**	**7-10**
**Supplementary Table 1. Summary of Key Discussion Points from Facilitated Discussions**	**11-12**
**Supplementary Table 2: Practical Considerations related to Digital Literacy, Accessibility and Funding of Telerehabilitation (TR)**	**13-14**

Supplementary Appendix 1: Telerehabilitation in solid organ transplantation in Canada: Celebrating achievements and designing the future

DAY 1: February 6, 2023 | 11 am - 4 pm EST

Time	Description	Speaker
11:00 am	Opening Remarks	Dmitry Rozenberg, Sunita Mathur & Sherrie Logan
11:15 am	Patient Story	Rienk de Vries
11:30 am	The evolving landscape of tele-rehabilitation: considerations for the solid organ transplant care continuum	Lisa Wickerson

11:50 PM | STRETCH BREAK

Delivery Methods – Rapid Fire Presentations		
11:55 am	Telerehabilitation in SOT recipients: effects of telerehabilitation on physical function, mental health and quality of life - A systematic review	Manoela Ferreira & Sandrine Juillard
12:10 pm	Reflections on delivering virtual exercise interventions in adults with cardiovascular disease	Jennifer Reed
12:20 pm	Conducting a Virtual Exercise Program for Pediatric Solid Organ Transplant Patients	Astrid De Souza
12:30 pm	Virtual home-based rehabilitation programs to treat sarcopenia in youth after SOT	Diana Mager
12:40 pm	Question period	

12:50 PM | STRETCH BREAK

1:00 pm	Breakout Session<br>Group discussion	Dmitry Rozenberg, Manoela Ferreira & Sherrie Logan & Leah Getchell

1:45 - 2:15 PM | LUNCH BREAK

User Experiences – Panel & Discussion		
2:15 pm	Patient partner experience with the virtual applications and exercise	Donna Hart
2:25 pm	Identifying the Desired Features of Digital Health Tools from a User-Centered Design Approach	Sunita Mathur
2:30 pm	A quality improvement process to optimize hybrid rehabilitation delivery for lung transplant candidates	Manoela Ferreira
2:35 pm	My Kidneys My Health: an eHealth tool co-created with patients, for patients	Mo Donald
2:40 pm	Group Discussion	Leah Getchell

3:00 PM | STRETCH BREAK

Future Directions & Priorities		
3:10 pm	Jamboard Brainstorm & Group discussions	Leah Getchell
3:50 pm	Closing Remarks	Dmitry Rozenberg, Sunita Mathur & Sherrie Logan

*All times are reported in EST/Toronto time.

DAY 2: February 10, 2023 | 11 am - 4 pm EST

Time	Description	Speaker
11:00 am	Opening Remarks	Dmitry Rozenberg, Sunita Mathur & Sherrie Logan
11:10 am	Pediatric Experience	Elaine Yong
11:20 am	Patient Story	Afsana Lallani
**Technological Consideration – Rapid Fire Presentations**		
11:30 am	Clinical and Research Delivery of Exercise Training	Dmitry Rozenberg
11:40 am	Technologies in the Liver Transplant Population	Puneeta Tandon
11:50 am	Safety and Special Considerations in the Virtual Exercise Training Environment	Tania Janaudis-Ferreira
12:00 pm	Janus face of virtualization – opportunities and challenges for tele-rehabilitation	Istvan Mucsi
12:10 pm	Question period	

12:20 PM | STRETCH BREAK

12:30 pm	Breakout Session<br>Group discussion	Dmitry Rozenberg, Sunita Mathur & Sherrie Logan & Leah Getchell

1:15 - 1:45 PM | LUNCH BREAK

Clinical Application using Available Tools– Panel & Discussion		
1:45 pm	Caregiver Perspective with Tele-Rehabilitation	Sherrie Logan
1:55 pm	Assessment tools to monitor changes in fitness and activity levels in pediatric patients	Anita Cote
2:00 pm	Tele-Rehabilitation through the Thoracic Transplant Journey: Acute Pediatric Considerations	Robin Deliva
2:05 pm	Patient's engagement needs for telerehabilitation	Nicholas Bourgeois
2:10 pm	Assessing Frailty in the Virtual Environment: Applications for Telerehabilitation	Karthik Tennankore
2:15 pm	Group Discussion	Leah Getchell

2:35 PM | STRETCH BREAK

Future Directions & Priorities		
2:45 pm	Jamboard Brainstorm & Group discussions	Leah Getchell
3:30 pm	Closing Remarks	Dmitry Rozenberg, Sunita Mathur & Sherrie Logan

*All times are reported in EST/Toronto time.

Supplementary Appendix 2: Methodological Approach to 2-Day Virtual Meeting

Meeting Participants:

Participants were invited through the Canadian Donation Transplant Research Program (CDTRP), which is a national organization advancing the interests of SOT recipients. Some participants previously expressed interest in the topic of exercise and physical fitness. We had good representation across Canadian transplant centers (Vancouver, Edmonton, Calgary, London, Toronto, Ottawa, Montreal, and Halifax), organ representation (i.e. heart, lung, kidney, and liver), adult and pediatric healthcare expertise, patients and caregiver participants, and varying experience with tele-rehabilitation (TR) across clinical and research settings including trainees. Participation in the meeting was voluntary and patient/caregiver participants were compensated for their time as per CDTRP policy on reimbursement.

Two-day Virtual Meeting Presentations and Discussion:

Each day consisted of scheduled presentations, a question period, one breakout session comprised of three different small group discussions, followed by a larger group discussion summarizing the dialog of the three groups, and one Google Jamboard brainstorm session at the end of the day, as shown in **Supplementary Appendix 1**. The two-day virtual meeting was facilitated by an experienced facilitator (LG) and all presentations and discussion were recorded through ZOOM.

Discussion topics included the barriers and facilitators to TR delivery, safety and special considerations with TR, digital literacy and privacy of TR, and physiologic and clinical outcome measures in patients participating in TR. Key questions posed during group discussions included the following: 1) What are the gaps as they relate to future clinical and research priorities in TR? 2) What are future research projects and paradigms to consider, as well as timelines in TR? and 3) What research funding opportunities are available? The discussions focused on exchange of ideas and consensus was not sought.

Summary of Presentations and Synthesis of Discussion:

The key discussion points were summarized by two members of the study team (SS, DR) to ensure summaries were reflective of the recorded group discussions and written jam board summaries. The synthesis and tables were then reviewed by meeting participants and co-authors and incorporated into the manuscript. A summary of the discussion topics and summary points are listed in **Supplementary Table 1**.

Supplementary Appendix 3:

Literature Search performed on June 24, 2024

Ovid MEDLINE(R) ALL <1946 to June 21, 2024>

#	Searches	Results	Type
1	[Solid Organ Transplantation]	0	Advanced
2	Organ Transplantation/	16375	Advanced
3	exp Heart Transplantation/	40441	Advanced
4	Kidney Transplantation/	107398	Advanced
5	Liver Transplantation/	66141	Advanced
6	exp Lung Transplantation/	19335	Advanced
7	Pancreas Transplantation/	7873	Advanced
8	Transplant Recipients/	8216	Advanced
9	Transplantation/	9021	Advanced
10	(organ? adj2 transplant*).mp.	45363	Advanced
11	(organ? adj2 graft*).mp.	1068	Advanced
12	(organ? adj2 allograft*).mp.	974	Advanced
13	(organ? adj2 allotransplant*).mp.	82	Advanced
14	(organ? adj2 heterograft*).mp.	1	Advanced
15	(organ? adj2 heterotransplant*).mp.	1	Advanced
16	(organ? adj2 homotransplant*).mp.	8	Advanced
17	(organ? adj2 homograft*).mp.	9	Advanced
18	(heart? adj2 transplant*).mp.	50717	Advanced
19	(heart? adj2 graft*).mp.	1326	Advanced
20	(heart? adj2 allograft*).mp.	1731	Advanced
21	(heart? adj2 allotransplant*).mp.	76	Advanced
22	(heart? adj2 heterograft*).mp.	17	Advanced
23	(heart? adj2 heterotransplant*).mp.	2	Advanced
24	(heart? adj2 homotransplant*).mp.	26	Advanced
25	(heart? adj2 homograft*).mp.	61	Advanced
26	(cardiac adj2 transplant*).mp.	12437	Advanced
27	(cardiac adj2 graft*).mp.	1203	Advanced
28	(cardiac adj2 allograft*).mp.	5726	Advanced
29	(cardiac adj2 allotransplant*).mp.	98	Advanced

30	(cardiac adj2 heterograft*).mp.	11	Advanced
31	(cardiac adj2 heterotransplant*).mp.	2	Advanced
32	(cardiac adj2 homotransplant*).mp.	13	Advanced
33	(cardiac adj2 homograft*).mp.	53	Advanced
34	(cardiothoracic adj2 transplant*).mp.	175	Advanced
35	(cardiothoracic adj2 graft*).mp.	3	Advanced
36	(cardiothoracic adj2 allograft*).mp.	1	Advanced
37	(cardiothoracic adj2 allotransplant*).mp.	0	Advanced
38	(cardiothoracic adj2 heterograft*).mp.	0	Advanced
39	(cardiothoracic adj2 heterotransplant*).mp.	0	Advanced
40	(cardiothoracic adj2 homotransplant*).mp.	0	Advanced
41	(cardiothoracic adj2 homograft*).mp.	0	Advanced
42	(cardiopulmonary adj2 transplant*).mp.	241	Advanced
43	(cardiopulmonary adj2 graft*).mp.	514	Advanced
44	(cardiopulmonary adj2 allograft*).mp.	3	Advanced
45	(cardiopulmonary adj2 allotransplant*).mp.	4	Advanced
46	(cardiopulmonary adj2 heterograft*).mp.	0	Advanced
47	(cardiopulmonary adj2 heterotransplant*).mp.	0	Advanced
48	(cardiopulmonary adj2 homotransplant*).mp.	2	Advanced
49	(cardiopulmonary adj2 homograft*).mp.	2	Advanced
50	(liver? adj2 transplant*).mp.	90797	Advanced
51	(liver? adj2 graft*).mp.	6335	Advanced
52	(liver? adj2 allograft*).mp.	2864	Advanced
53	(liver? adj2 allotransplant*).mp.	88	Advanced
54	(liver? adj2 heterograft*).mp.	2	Advanced
55	(liver? adj2 heterotransplant*).mp.	3	Advanced
56	(liver? adj2 homotransplant*).mp.	38	Advanced
57	(liver? adj2 homograft*).mp.	39	Advanced
58	(hepat* adj2 transplant*).mp.	8393	Advanced
59	(hepat* adj2 graft*).mp.	972	Advanced
60	(hepat* adj2 allograft*).mp.	621	Advanced
61	(hepat* adj2 allotransplant*).mp.	24	Advanced
62	(hepat* adj2 heterograft*).mp.	0	Advanced
63	(hepat* adj2 heterotransplant*).mp.	5	Advanced
64	(hepat* adj2 homotransplant*).mp.	9	Advanced
65	(hepat* adj2 homograft*).mp.	11	Advanced

66	(pancrea* adj2 transplant*).mp.	10972	Advanced
67	(pancrea* adj2 graft*).mp.	1913	Advanced
68	(pancrea* adj2 allograft*).mp.	1123	Advanced
69	(pancrea* adj2 allotransplant*).mp.	176	Advanced
70	(pancrea* adj2 heterograft*).mp.	1	Advanced
71	(pancrea* adj2 heterotransplant*).mp.	1	Advanced
72	(pancrea* adj2 homotransplant*).mp.	14	Advanced
73	(pancrea* adj2 homograft*).mp.	9	Advanced
74	(lung? adj2 transplant*).mp.	26813	Advanced
75	(lung? adj2 graft*).mp.	853	Advanced
76	(lung? adj2 allograft*).mp.	2373	Advanced
77	(lung? adj2 allotransplant*).mp.	215	Advanced
78	(lung? adj2 heterograft*).mp.	0	Advanced
79	(lung? adj2 heterotransplant*).mp.	7	Advanced
80	(lung? adj2 homotransplant*).mp.	26	Advanced
81	(lung? adj2 homograft*).mp.	10	Advanced
82	(thoracic adj2 transplant*).mp.	588	Advanced
83	(thoracic adj2 graft*).mp.	1698	Advanced
84	(thoracic adj2 allograft*).mp.	17	Advanced
85	(thoracic adj2 allotransplant*).mp.	2	Advanced
86	(thoracic adj2 heterograft*).mp.	0	Advanced
87	(thoracic adj2 heterotransplant*).mp.	0	Advanced
88	(thoracic adj2 homotransplant*).mp.	0	Advanced
89	(thoracic adj2 homograft*).mp.	15	Advanced
90	(pulmonary adj2 transplant*).mp.	1196	Advanced
91	(pulmonary adj2 graft*).mp.	417	Advanced
92	(pulmonary adj2 allograft*).mp.	353	Advanced
93	(pulmonary adj2 allotransplant*).mp.	15	Advanced
94	(pulmonary adj2 heterograft*).mp.	10	Advanced
95	(pulmonary adj2 heterotransplant*).mp.	2	Advanced
96	(pulmonary adj2 homotransplant*).mp.	13	Advanced
97	(pulmonary adj2 homograft*).mp.	520	Advanced
98	(kidney? adj2 transplant*).mp.	122462	Advanced
99	(kidney? adj2 graft*).mp.	5499	Advanced
100	(kidney? adj2 allograft*).mp.	5191	Advanced

101	(kidney? adj2 allotransplant*).mp.	243	Advanced
102	(kidney? adj2 heterograft*).mp.	2	Advanced
103	(kidney? adj2 heterotransplant*).mp.	3	Advanced
104	(kidney? adj2 homotransplant*).mp.	108	Advanced
105	(kidney? adj2 homograft*).mp.	48	Advanced
106	(renal adj2 transplant*).mp.	52220	Advanced
107	(renal adj2 graft*).mp.	4176	Advanced
108	(renal adj2 allograft*).mp.	13336	Advanced
109	(renal adj2 allotransplant*).mp.	457	Advanced
110	(renal adj2 heterograft*).mp.	3	Advanced
111	(renal adj2 heterotransplant*).mp.	19	Advanced
112	(renal adj2 homotransplant*).mp.	335	Advanced
113	(renal adj2 homograft*).mp.	251	Advanced
114	or/2-113	336267	Advanced
115	rh.fs.	210620	Advanced
116	exp Rehabilitation/	364477	Advanced
117	rehab*.mp.	393969	Advanced
118	telerehab*.mp.	2789	Advanced
119	115 or 116 or 117 or 118	641840	Advanced
120	114 and 119	3814	Advanced
121	limit 120 to english language	3343	Advanced
122	limit 121 to "review articles"	626	Advanced
123	Cross-Sectional Studies/	506192	Advanced
124	exp Cohort Studies/	2620063	Advanced
125	123 or 124	3032595	Advanced
126	121 and 125	980	Advanced
127	122 or 126	1580	Advanced
128	limit 127 to yr="2004 -Current"	1048	Advanced
129	remove duplicates from 128	1048	Advanced

Supplementary Table 1:
Summary of Key Discussion Points from Facilitated Discussions

Discussion Topics	Summary of Key Discussion Points
Barriers to Telerehabilitation	• Patient safety concerns<br>• Technology/equipment requirements for patients and/or healthcare providers<br>• Language barriers<br>• Patient interest<br>• Programs may not be individualized<br>• Lack of funding and staffing
Facilitators to Telerehabilitation	• Providing rehab initially in-person, followed by telerehabilitation may help facilitate learning<br>• Having a strong team to build rapport with patients<br>• Tailoring program to meet individual needs<br>• Having a transplant mentor<br>• Telerehabilitation can increase accessibility for patients (e.g. remote populations, lack of transportation)<br>• Patient caregivers<br>• Developing safety protocols and guidelines<br>• Having educational sessions
Safety and special considerations with telerehabilitation delivery	• Having initial baseline assessments in-person<br>• Oxygen needs for patients with lung disease<br>• Mental health considerations for patients<br>• Patient safety and preferences should be individualized<br>• When should telerehabilitation be initiated and completed in the transplant journey?
Digital literacy and privacy of telerehabilitation	• Access may be challenging for patients with low digital literacy<br>• For patients with cognitive impairment, caregiver may be able to help<br>• Using encryption or institution approved platforms to ensure patient confidentiality
Physiologic and clinical outcome measures in patients participating in telerehabilitation	• Might be challenging for patients to reach target heart rates with telerehabilitation

What are the gaps as they relate to future clinical and research priorities?	• Safety and virtual monitoring of telerehabilitation, and integration with electronic records<br>• Ability to share resources/experiences across centers<br>• Mental health considerations for patients and caregivers
What are future research projects and paradigms to consider, as well as timelines?	• Creating standardized safety protocols and guidelines<br>• Developing flexible and individualized programs<br>• Virtual reality or artificial intelligence based telerehabilitation programs<br>• Research project on mental health
What are some funding opportunities available?	• Canadian Institutes of Health Research<br>• Canadian Donation and Transplantation Research Program (CDTRP)<br>• Canadian Society of Transplantation (CST)<br>• National Institutes of Health<br>• American Society of Transplantation<br>• International Society of Heart and Lung Transplantation

Supplementary Table 2:

Practical Considerations related to Digital Literacy, Accessibility and Funding of Telerehabilitation (TR)

Barriers	Practical Recommendations
**Digital Literacy**	▪ Organizations to develop education on TR platforms that are publically available for other institutions<br>▪ Patient and caregiver educational resources publically available; developed by health care organizations and health policy funders to promote sustainability<br>▪ Availability of technological support for health care providers, patients and caregivers
**Accessibility**	▪ Provide electronic tablets for patients without access<br>▪ Accessible physical space to utilize TR for both health care providers and patients/caregivers;<br>▪ Satellite centers close to home for patients without access to internet or technology
**Funding**	▪ Need to demonstrate efficacy of TR programs<br>▪ Evaluation of cost-effectiveness of TR and various models of TR delivery (i.e. virtual vs. center-based programs).<br>▪ Efficacy of TR programs on clinical outcomes such as health care utilization (i.e. hospitalizations, physician visits)
